# Workplace physical activity practices in real life: a scoping review of grey literature for small- and medium-sized enterprises

**DOI:** 10.1093/eurpub/ckac083

**Published:** 2022-08-26

**Authors:** Ilkka Väänänen, Sebastià Mas-Alòs, Frank Vandaele, Anna Codina-Nadal, Sergi Matas, Eva Aumatell, Ine De Clerk, Anna Puig-Ribera

**Affiliations:** LAB University of Applied Sciences, Health Care Unit, Physical Activity and Functional Capacity Research Group, Lahti Campus, Finland; National Institute of Physical Education of Catalonia (INEFC), Lleida Campus, Catalonia, Spain; University of Lleida (UdL), Human Movement Research Group, Catalonia, Spain; Artevelde University of Applied Sciences, Gent, Belgium; University of Vic-Central University of Catalonia, Centre for Health and Social Care Research, Sport and Physical Activity Research Group, Vic, Spain; National Institute of Physical Education of Catalonia (INEFC), Lleida Campus, Catalonia, Spain; University of Lleida (UdL), Human Movement Research Group, Catalonia, Spain; Open University of Catalonia, eHealth Center, Barcelona, Spain; Artevelde University of Applied Sciences, Gent, Belgium; University of Vic-Central University of Catalonia, Centre for Health and Social Care Research, Sport and Physical Activity Research Group, Vic, Spain

## Abstract

**Background:**

There is a need to scale-up effective physical activity (PA) programmes for small- and medium-sized enterprises (SMEs), where the uptake of PA interventions is low. Identifying real-life workplace practices in PA could contribute to a better understanding of what PA programmes might be most grounded in the ‘real world’. However, there is a scarcity of evidence showing what gets done. This study aimed to identify, describe and comprehensively summarize the real-life implementations of workplace PA initiatives, particularly in Europe, as a prior step to disseminating future feasible PA practices for SMEs.

**Methods:**

A scoping review of grey literature included a systematic search in the Google advanced search platform that permuted a combination of key concepts (PA, workplace, interventions/programmes), internet domains, and search operators in six different languages (Catalan, Finnish, French, Dutch, English and Spanish) between 2015 and November 2020. The analysis process was iterative, and multiple methods were used to sort, group and categorize the initiatives.

**Results:**

There were a total of 713 real-life workplace PA initiatives from different-sized organizations identified. These were categorized into five themes: active work and living, exercise and fitness programs, management and leadership, communication and dissemination, and facilities. Finally, feature trees showing a menu for real-life workplace PA practices were implemented.

**Conclusions:**

Identifying real-life practice providing a state-of-the-art snapshot of current PA practices in workplaces, which is a starting point to better understand feasible practices in the context of small- and medium-sized workplaces.

## Introduction

Physical inactivity is associated with an increased risk of premature mortality and non-communicable diseases such as coronary heart disease, type 2 diabetes and breast and colon cancer.^1^ Regular physical activity (PA) also benefits sleep quality, executive function, cognition, mental health, perceived quality of life^2^ and work productivity.^3^ However, 4 in 10 Europeans spend an average of 5 and a half hours or more sitting down each day.^4^ In 2020, more than one in three adults of 28 European Union member states, as well as the UK, were physically inactive,^5^ and thus not meeting the World Health Organization’s (WHO) recommendations on PA and sedentary behaviour.^6^ In this context, there is a need to increase populations’ PA and make it part of everyone’s day-to-day life.^7^

Tackling sedentary and physically inactive lifestyles in all age groups is a universal challenge that requires system-wide multisectoral efforts. In 2018, the WHO published the Global Action Plan for PA 2030,^8^ providing 20 policies across multiple settings to promote PA as a whole system approach. The workplace is one of these fundamental settings for the potential it has to reach much of the adult population.^9^ On this basis, a package of practical implementation guidelines for effective PA interventions^10^ highlighted workplaces as key platforms to provide all adults with opportunities and counselling for PA (Policy action area 3: Active people; Action 3.3). Most importantly, the PA strategy for the WHO European Region 2016–25^11^ emphasized the essential role workplaces have for reaching all employees, including those from the informal sector, socially disadvantaged groups, women and manual workers (Priority area 3, Objective 3.2, Action 42).

This strategy is a considerable challenge that depends on the scalability of effective, feasible and sustainable PA programmes in the majority of workplaces. While scale-up of effective PA interventions across settings has been uneven,^7^ in European workplaces insufficient scaling-up is a key issue in a context where a significant proportion of workplaces are small- and medium-sized enterprises (SMEs) which experience greater difficulties when implementing PA programmes.^9^

On the one hand, sufficient evidence from systematic reviews exists to identify PA promotion programmes that might be effective in workplaces.^12–25^ However, the relevance of systematic reviews for increasing the possibilities of the uptake of PA programmes is unclear.^26^ While systematic reviews indicate what is effective and how much the effect size is—a critical factor in deciding whether programmes to be scaled up might be a good investment—they do not take into account the context of workplaces suggested by the social–ecological model.^27^^,^^28^ Therefore, systematic reviews might not identify the essential PA programme elements that are needed for a broad-scale application of effective PA programmes to specific occupational contexts.

Yet, on the other hand, there is a scarcity of evidence that identifies what gets implemented in real life. However, identifying real-life workplace practices in PA could contribute to understanding what PA programmes might be most grounded in the ‘real world’. Although identifying best practices for occupational PA interventions should be based on the application of principles of programme design, evaluation and multicomponent system approaches,^27^ this might be a cost-effective step prior to embarking on more extensive stakeholder engagement for developing practical guidelines to promote PA in SMEs. Importantly, identifying real-life practice allows a better understanding of the contexts where workplace PA programmes take place, which might be a key issue before disseminating future best practices of PA interventions across SMEs.

Based on the need to bridge the gap between research and practice, the purpose of this scoping review was to identify, describe and comprehensively summarize real-life implementations of workplace PA initiatives, particularly in Europe. Findings from this ‘real world’ formative research will be the basis for exploring the perceived most feasible PA practices to be implemented in the context of smaller workplaces.

## Methods

Scoping reviews are an ideal tool to provide an overview of ‘real world’ PA implementations in workplaces and identify key characteristics to inform practices on what has been conducted.^29^ The scoping review was carried out according to the suggested framework^30^ and the PRISMA checklist^31^ for assessing quality criteria. As a result of the reviewed evidence, a database showing a menu of choices for real-life workplace PA practices was developed.

The grey literature search strategy for the scoping review included a Web search with Google that responded to the research question ‘Which real-life PA initiatives do workplaces for industry or business promote to employees?’ The research question was formulated using the SPICE framework, a structured approach that constructs practice questions based on Setting (where? in what context?), Perspective (from whom?), Intervention (what?), Comparison (what else?) and Evaluation (how well? what result?).^32^

### Google search strategy

Evidence from grey literature on real-life workplace PA practice was identified from the advanced search platform for Google (https://www.google.es/advanced_search) by permuting a combination of key concepts (PA, workplace, interventions/programmes), internet domains and search operators in six different languages (English, French, Spanish, Finnish, Dutch and Catalan) between 2015 and November 2020. This search randomly permuted a combination of terms in the key dimensions set according to the SPICE framework and the Statistical Classification of Economic Activities in the European Commission which included: (i) workplace intervention or campaign aiming to increase PA, physical fitness, exercise or reduce sedentary behaviours, (ii) intervention initiated, implemented or organized by employers or workplaces and (iii) PA, physical fitness or sedentary behaviour outcomes.

To identify specific key terms within each dimension, an initial search combining these dimensions was carried out using the following English terms: workplace, workers, employees, staff, programme, promotion and PA. The first 10 results for relevant documents were analyzed and keywords within each dimension were added to the search strategy. Then, several search strategies using the added keywords from each dimension were implemented to identify the search strategy that yielded the best results.

#### Selection of documents/resources from the google search strategy

The search for grey literature included any resources produced by organizations and businesses outside the traditional scientific and academic distribution channels such as reports (annual, research, technical and project reports), working papers, document papers, white papers, evaluations and web-based information produced by industry, business, international organizations, national or local governments. The search strategy was limited to resources published during 2015–20 and files in pdf or html format.

From the search strategies in each language, the first 100 results of relevant records were cross-checked. Relevant records tend to appear in the first ten pages according to the Google search criteria (https://support.google.com/websearch/answer/2466433?hl=en).

All the Google records shown as relevant in the Google algorithm for Catalan, Dutch, Finnish, French, English and Spanish were reviewed by titles and nomenclature by native researchers (*n* = 2 for each language). Relevant records were considered fully eligible if they helped to answer the research question. Such records were included in the final comprehensive synthesis from which relevant files were analyzed. Any disagreement about document eligibility was resolved for each language by other authors. The full text of eligible records illustrated workplace PA programmes that were currently implemented, had been implemented in real life or were intended to be implemented. The flowchart diagrams (PRISMA 2020, https://prisma-statement.org/PRISMAStatement/FlowDiagram) that map out the number of records identified, included, and excluded in the different phases of the Google search are available in Supplementary appendix 1.

#### Data extraction and theoretical integration of the literature

Data were systematically extracted from the selected documents and resources: (a) initiative, (b) country, (c) name of enterprise, (d) partners, (e) industry or business, (f) areas of focus, (g) brief description of the initiative, (h) timeline, (i) links, (j) number or % of participants/employees and (k) results of the evaluation. Regarding areas of focus (f), the following details were extracted according to nine categories identified from previous systematic reviews: (i) time of delivery and location, (ii) sectors, (iii) target behaviour and intensity, (iv) type/mode of activity, (v) target employees, (vi) level of impact, (vii) who delivers it, (viii) programme characteristics and (ix) existence of evaluation outcomes.^12–25^ The full description for each area of focus is presented in Supplementary appendix 2.

#### Data analysis

Multiple data analysis methods were used in this study. First, the individual data of the initiatives from each language were translated and merged together into one English raw data file. This first preliminary database included a total of 778 workplace PA initiatives, from which 22 resulted in being duplicates found several times across the different language searches, leading to a second preliminary database of 756 workplace PA initiatives (*n* = 701 for the English, *n* = 16 for the Dutch, *n* = 6 for the Finnish, *n* = 21 for the French, *n* = 3 for the Catalan and *n* = 9 for the Spanish searches). Thereafter, word clouds from the brief descriptions were drawn to illustrate the spectrum of the initiatives. The data represented initiatives from 14 European countries, America, Australia, Canada and Japan (22%). Most of the initiatives (79%) were from multisectoral workplaces, while the rest were from manufacturing or industrial-based (7%), office-based (7%), schools (2%), services-based (2%) and from other sectors (3%). Initiatives were focused on employees, half of whom (51%) were identified from governmental documents. The special group of employees at high risk or with poor health was mentioned in only 15% of the initiatives.

In order to develop the preliminary Workplace Physical Activity menu of choices and to provide stakeholders with programmes that could be used to promote PA to employees in SMEs, a second phase for data analysis was performed. This next step consisted of conducting a theory-based abductive thematic content analysis, which emphasizes the close interactive relationship between theory and data.^33^ First, all workplace PA initiatives from the second preliminary database were read through several times. After this, the initiatives were qualitatively organized into the main categories as described in the area of focus ‘Target behaviour & Intensity’. Based on these data, the feature themes and subclasses of the initiatives were summarized by one researcher (*n* = 111 were identified for sedentary behaviour-related initiatives; *n* = 237 for exercise and fitness programmes, *n* = 182 for active living at a light intensity, *n* = 67 for active living of at least moderate and, *n* = 159 were considered ‘no focus’ since these were not classified within any of these categories). The ‘no focus’ categorized initiatives were cross-checked independently by two researchers, who jointly decided how to reclassify them into the following types of target behaviour categories: Active travel, Facilities, Dissemination and Implementation, Management and Leadership and Assessments and Measurements. These initiatives were classified by analyzing them for similarities and identifying the semantic relationships of the descriptions. Then, they were sorted, grouped and named within the terms describing them. In this way, the data were condensed and clarified.

Finally, the workplace PA initiatives included within the other ‘Target behaviour & intensity’ categories were cross-checked independently again by two researchers in order to identify whether they would fit better being reallocated into the new categories. This process led to a third preliminary database of workplace PA initiatives categorized as follows: Active travel (*n* = 20), Facilities (*n* = 51), Dissemination and Implementation (*n* = 71), Management and Leadership (*n* = 84), Exercise programmes and fitness (*n* = 238), Sedentary behaviour-related initiatives (*n* = 81), Active living at light intensity (*n* = 150) and Active living of at least moderate intensity (*n* = 18). During this process, another 43 initiatives were excluded from the database because they (i) were repeated initiatives that had been initially listed in different categories or (ii) had no link available to cross-check for information. Thus, a total of 713 were included in the final database as a menu of choices for SMEs to promote PA. From this final database, the tables of themes and their subclasses (see the Supplementary material) were developed, the resultant text was written and feature tree figures were drawn. The analysis process was iterative in nature, and the research group acted as ‘critical friends’^35^ during its different stages.

### Data availability

The data underlying this article are available online as Supplementary material.

## Results

### Description of the workplace PA initiatives

**Figure 1:**
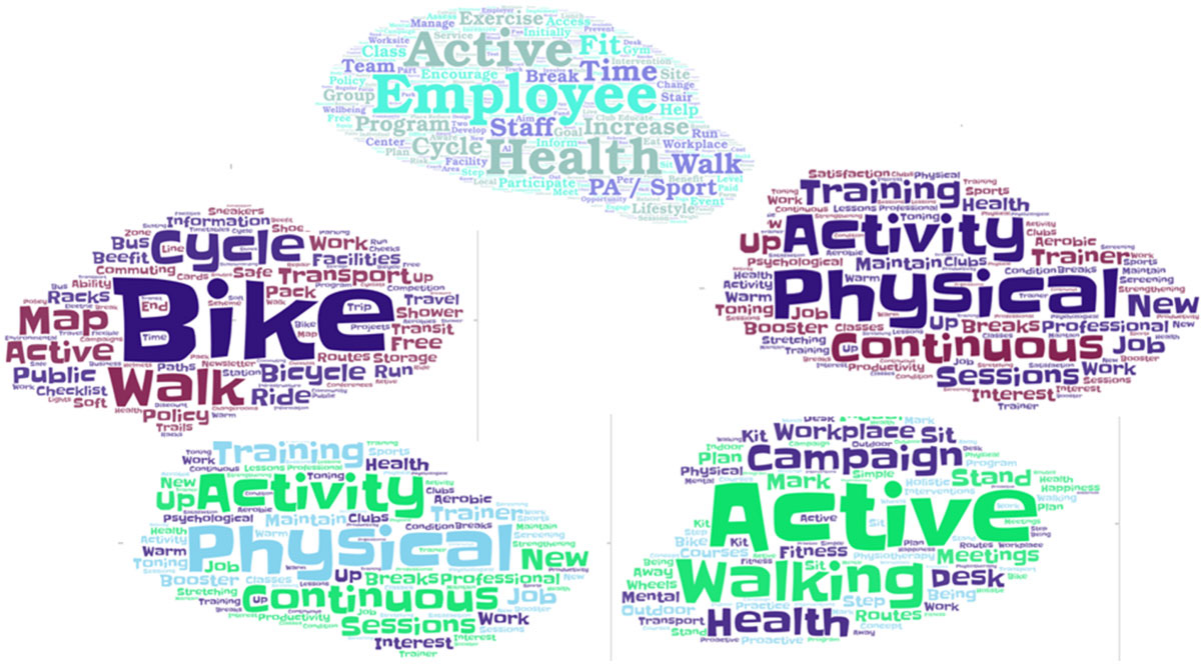
The word art spectrum of the initiatives of PA in SMEs

The brief descriptions of the initiatives included 20 333 words, and the spectrum of the initiatives is illustrated in the word clouds in figure 1. These words were included in the menu of choices for the identified work-based PA programmes.

### Content of the workplace PA initiatives

Workplace PA initiatives included both micro- and macro-level initiatives which were classified by the content into the following five different categories: Active work and living (*n* = 299); Exercise and fitness programmes (*n* = 238); Management and leadership (*n* = 84); Communication and dissemination (*n* = 71); and Facilities (*n* = 51). Most of the initiatives were focused on either active work and living (38%), or exercise and fitness programmes (33%). The rest were in smaller portions (ranging between 7% and 12%).

The themes, subclasses and description of initiatives for each category are presented in the Supplementary material. The details of each category are presented in the results section and are described along with the original initiatives.

#### Active work and living

There were three themes (Work, Active Travel, and Active Living) identified from the initiatives (*n* = 299) of the Active work and living category. The work theme included the tuning of the workstation, where ergonomics, sit-stand desks and flexible work schedules were the used initiatives. In addition, the follow-up, and activating subclasses included in the work theme. The active travel theme comprised a walkability and a cycling subclass, covering initiatives geared at increasing walking and the use of bicycles for commuting to work. Moreover, the active living theme consisted of activating initiatives such as the use of stairs, different activity campaigns and events, challenges, and competitions to activate time outside working hours.

#### Exercise and fitness programmes

One-third of all initiatives (*n* = 238) included the Exercise and fitness programmes category, from which three themes were identified: Programmes, Training, and Healthcare services. The programmes included long-lasting counselling and coaching interventions, and measurements before and after the programmes. The training theme included different types of sessions and activities such as cardiovascular resistance, strength, balance and flexibility sessions, and mental exercises, outside the workplace. In addition, games and boot camps were classified into this subclass. The third subclass in the programme theme was healthcare services where different professional healthcare services such as physiotherapy, and health assessments were produced.

#### Management and leadership

In the category of Management and leadership (*n* = 84), two themes (Organizational, and Individual level) of initiatives were identified. The organization level included the three subclasses of Documentation, Evaluation/Checking, and Healthcare. The initiatives of the documentation of the management included Human Resources policies and action plans, and their evaluation methods and instruments. Being included in the preventive healthcare system was expressed to be one of the initiatives. In the individual theme, the two identified subclasses were the bonus system and the culture of encouragement. The initiatives of the bonus system were either financial or other material benefits, and the workplace action culture was based on the acceptance and commitment of supporting PA and providing individual behaviour change strategies.

#### Communication and dissemination

In the category of Communication and dissemination (*n* = 71), two themes of initiatives were identified. Communication included educational, informational and reflective subclasses, where conferences, seminars and lectures were the initiatives used to educate the employees. The information was shared at expos and at information desks. Only workshops were mentioned as participatory initiatives. Different dissemination materials and channels were found as tools to inform the staff.

#### Facilities

There were both supplies and premises in the Facilities category (*n* = 51). The supplies subclass included both storage and equipment, where items for cycling were mentioned as storage initiatives. Typical initiatives in the other subclass of facilities were the supply of sports equipment and subsidizing this. Sports facilities, and dressing and washing rooms were included in the premises theme.

## Discussion

The purpose of this qualitative descriptive study was to analyze, describe and comprehensively summarize real-life implementations of workplace PA initiatives. Data were collected from grey literature published in the advanced Google search platform using the scoping review methods. Workplace PA initiatives were classified into the following five different categories: Active work and living; Exercise and fitness programmes; Management and leadership; Communication and dissemination; and Facilities. These initiatives included both organizational and individual level practices from over seven hundred real-life PA initiatives analyzed qualitatively by the content.

Most initiatives were focused on activating either the work or daily lives of the employees. The Exercise and fitness programmes, and Management and leadership themes included organization and employee level initiatives. In contrast, the Communication and dissemination theme was focused on providing education and information to the employees rather than promoting employee-based reflection. The facilities theme was formed from elements of the infrastructural and requisite framework. All these initiatives implemented a Workplace Physical Activity Menu of Choices grounded in the ‘real world’. To our knowledge, this is the first study that provides a state-of-the-art snapshot of current PA practices in workplaces. As part of a series of two studies, the findings from this ‘real world’ formative research will be the basis to explore the perceived most feasible PA practices to be implemented in the context of smaller workplaces. Then, effective PA practices for SMEs should be identified according to specific principles of programming and recommendations for practice based on an evidence approach.^27^

This scoping review study provided an extensive snapshot of the efforts made to promote PA in workplaces. A large number of initiatives were recognized which clearly demonstrates the significant input of promoting employee PA in workplaces through a wide range of initiatives. However, workplaces seemed to mainly implement practices belonging to one single category without being attached to other categories, especially to initiatives involving the organization's strategic KPIs. Therefore, a lack of system-wide multisectoral efforts and attempts to promote PA as a part of the multicomponent framework of cost-effective workplaces seemed to be identified. For example, though health promotion was mentioned in initiatives, social cohesion or the value chain to the organization or society was not referred to as much. In addition, there seemed to be a lack of promoting PA to individuals with diverse abilities to do PA. Furthermore, scarce evaluation inputs were identified from the implementation of these PA initiatives. In this context, this review could not identify which choices for a workplace PA were effective or could be considered best practices. Nonetheless, all these initiatives were grounded in the ‘real world’, which may imply that workplaces that implemented them had considered them feasible. Therefore, the practitioners can use the menu and its categories in planning the choices of appropriate workplace PA interventions at both micro- and macro-levels in organizations. This existing body of programmes can be used as a point of departure for developing future multi-component PA initiatives tailored to the specific context of SMEs. In order to obtain a comprehensive supply, they should choose at least one initiative from each subclass from each theme. Regarding the need to obtain a return on investment, future research should focus on making these ‘real-life’ PA interventions more effective to improve the economical, societal and value-added benefits for workplaces (e.g. improving social inclusion and effects according to age and gender). In a systemic model, researchers should take a holistic approach to PA initiatives by not only targeting individual behaviour change but also the complex and multifactorial existing factors^36^ of smaller enterprises if these are to be successfully scaled up to other SMEs.

This study has several strengths and limitations. The main strength was the review of grey literature that contained information not often available in academic or scientific documents. This was a key issue when trying to identify real-life workplace PA initiatives that do not belong to the implementation of scientific-funded projects. Although alternative methods could have been employed, such as surveys or expert interviews, these could have not identified such a broad range of PA initiatives at a global and European level. In the planned second part of this study, we intend to use a Delphi survey to identify barriers to implementing the menu of real-life PA initiatives in SMEs.

Although there is no accepted, gold standard method, and only little specific guidance is available for performing grey literature searches,^37^ several issues were taken into account to maximize search sensitivity and precision while keeping the search results manageable. These included: (i) using controlled vocabulary search, and collecting related words and index terms to specify possible related terms; (ii) expanding the search to more than one language, including the most spoken languages in Europe, to address the geographic and location-based features of the Google search; (iii) having a clear research question to be answered to manage the variability of documents identified; (iv) having clear criteria, from systematic reviews, to extract data from the selected documents and (v) having continuous meetings with the research team to cross-check findings and data extraction procedures across languages. Nonetheless, some difficulties of reviewing grey literature from web-based platforms could not be avoided such as some links and documents becoming unavailable after the initial search had taken place.^37^ However, it should be highlighted that the aim of this scoping review was to understand what is implemented in real life. We did not aim to identify every single workplace PA initiative currently implemented but to have most types of initiatives listed as a menu of choices of what can be done. In this scoping review of grey literature, six different European languages were chosen by the specific guidance available for performing grey literature searches:^37^ While it was not possible to fully meet all requirements with the limited resources available, we were able to (a) expand the search to more than one language (Catalan, Dutch, English, Finnish, French and Spanish), (b) include three of the most frequently spoken languages in Europe (English, French and Spanish) and (c) address European geographical features (northern: Finnish; middle: Dutch, English and French; southern: Catalan and Spanish). For the sake of comparability, the same absolute number of results were screened for each language, although search hits (i.e. the number of initiatives found) in the different languages varied. This approach was chosen based on the saturation principle in qualitative data collection. The main advantages of this approach are its sufficient and time-efficient amount of information control, location-based equality and conformity to the geography of the continent. On the other hand, possible disadvantages of this approach are the risks of limited information and the lack of rare findings.

To summarize, a database showing a menu of choices for real-life workplace PA practices was developed. Identifying real-life practice provided a state-of-the-art snapshot of current PA practices in workplaces, which is a starting point to better understand feasible practices in the context of small- and medium-sized workplaces. The absence of evidence on the evaluation of initiatives does not necessarily imply the absence of effects. Nevertheless, future research should aim to assess the effects of these real-life practices on workplace PA and focus on evaluating their effectiveness and scalability for boarder use in work communities.

## Supplementary data

Supplementary data are available at *EURPUB* online.

## Supplementary Material

ckac083_Supplementary_Data

## References

[1] Lee IM , Shiroma EJ, Lobelo F, et al; Lancet Physical Activity Series Working Group. Effect of physical inactivity on major non-communicable diseases worldwide: an analysis of burden of disease and life expectancy. Lancet 2012;380:219–29. 22818936 10.1016/S0140-6736(12)61031-9 PMC3645500

[2] King AC , Powell KE. 2018 Physical Activity Guidelines Advisory Committee Scientific Report. Washington, DC: U.S. Department of Health and Human Services, 2018.

[3] Hafner M , Yerushalmi E, Stepanek M, et al Estimating the global economic benefits of physically active populations over 30 years (2020-2050). Br J Sports Med 2020;54:1482–7. 33239354 10.1136/bjsports-2020-102590 PMC7719903

[4] European Commission. *Special Eurobarometer 472. Sport and Physical Activity.* 2017. Available at: https://data.europa.eu/doi/10.2766/599562 (26 June 2022, date last accessed).

[5] Nikitara K , Odani S, Demenagas N, et al Prevalence and correlates of physical inactivity in adults across 28 European countries. Eur J Public Health 2021;31:840–5. 34000007 10.1093/eurpub/ckab067 PMC8504996

[6] Bull FC , Al-Ansari SS, Biddle S, et al World Health Organization 2020 guidelines on physical activity and sedentary behaviour. Br J Sports Med 2020;54:1451–62. 33239350 10.1136/bjsports-2020-102955 PMC7719906

[7] The Lancet (editorial). A sporting chance: physical activity as part of everyday life. The Lancet 2021;398:365. 10.1016/S0140-6736(21)01652-4 34302762

[8] World Health Organization. Global action plan on physical activity 2018–2030: more active people for a healthier world. 2018. Available at: https://apps.who.int/iris/handle/10665/272722 (26 June 2022, date last accessed).

[9] Kite J , Chau J, Engelen L, Bellew B. The workplace domain and physical activity. In: Bellew B, Nau T, Smith B, Bauman A., editors. Getting Australia Active III. A Systems Approach to Physical Activity for Policy Makers. The Australian Prevention Partnership Centre and the University of Sydney, 2020.

[10] World Health Organization. *ACTIVE: A Technical Package for Increasing Physical Activity.* Geneva: World Health Organization, 2018. Licence: CC BY-NC-SA 3.0 IGO.

[11] World Health Organization Regional Office for Europe. Physical Activity Strategy for the WHO European Region 2016-2025. Copenhagen: World Health Organization, 2016.

[12] Abdin S , Welch RK, Byron-Daniel J, Meyrick J. The effectiveness of physical activity interventions in improving well-being across office-based workplace settings: a systematic review. Public Health 2018;160:70–6. 29751224 10.1016/j.puhe.2018.03.029

[13] Loitz CC , Potter RJ, Walker JL, et al The effectiveness of workplace interventions to increase physical activity and decrease sedentary behaviour in adults: protocol for a systematic review. Syst Rev 2015;4:178. 26653146 10.1186/s13643-015-0166-4 PMC4676873

[14] Wolfenden L , Goldman S, Stacey FG, et al Strategies to improve the implementation of workplace-based policies or practices targeting tobacco, alcohol, diet, physical activity and obesity. Cochrane Database Syst Rev 2018;11:CD012439. 30480770 10.1002/14651858.CD012439.pub2 PMC6362433

[15] Buckingham SA , Williams AJ, Morrissey K, et al Mobile health interventions to promote physical activity and reduce sedentary behaviour in the workplace: a systematic review. Digit Health 2019;5:2055207619839883. 30944728 10.1177/2055207619839883 PMC6437332

[16] Johnson S , Regnaux JP, Marck A, et al Understanding how outcomes are measured in workplace physical activity interventions: a scoping review. BMC Public Health 2018;18:1064. 30144823 10.1186/s12889-018-5980-x PMC6109358

[17] Jirathananuwat A , Pongpirul K. Promoting physical activity in the workplace: a systematic meta-review. J Occup Health 2017;59:385–93. 28740029 10.1539/joh.16-0245-RA PMC5635147

[18] White MI , Dionne CE, Wärje O, et al Physical activity and exercise interventions in the workplace impacting work outcomes: a stakeholder-centered best evidence synthesis of systematic reviews. Int J Occup Environ Med 2016;7:61–74. 27112715 10.15171/ijoem.2016.739 PMC6816510

[19] Burn NL , Weston M, Maguire N, et al Effects of workplace-based physical activity interventions on cardiorespiratory fitness: a systematic review and meta-analysis of controlled trials. Sports Med 2019;49:1255–74. 31115827 10.1007/s40279-019-01125-6

[20] Grimani A , Aboagye E, Kwak L. The effectiveness of workplace nutrition and physical activity interventions in improving productivity, work performance and workability: a systematic review. BMC Public Health 2019;19:1676. 31830955 10.1186/s12889-019-8033-1 PMC6909496

[21] MacEwen BT , MacDonald DJ, Burr JF. A systematic review of standing and treadmill desks in the workplace. Prev Med 2015;70:50–8. 25448843 10.1016/j.ypmed.2014.11.011

[22] Garne-Dalgaard A , Mann S, Bredahl TVG, Stochkendahl MJ. Implementation strategies, and barriers and facilitators for implementation of physical activity at work: a scoping review. Chiropr Man Therap 2019;27:48. 10.1186/s12998-019-0268-5 PMC6784342 31624537

[23] Muir SD , Silva SSM, Woldegiorgis MA, et al Predictors of success of workplace physical activity interventions: a systematic review. J Phys Act Health 2019;16:647–56. 31203701 10.1123/jpah.2018-0077

[24] Shrestha N , Kukkonen-Harjula KT, Verbeek JH, et al Workplace interventions for reducing sitting at work. Cochrane Database Syst Rev 2018;6:CD010912. 29926475 10.1002/14651858.CD010912.pub4 PMC6513236

[25] Kahn-Marshall JL , Gallant MP. Making healthy behaviours the easy choice for employees: a review of the literature on environmental and policy changes in worksite health promotion. Health Educ Behav 2012;39:752–76. 22872583 10.1177/1090198111434153

[26] Hubbard G , Thompson CW, Locke R, et al Co-production of "nature walks for wellbeing" public health intervention for people with severe mental illness: use of theory and practical know-how. BMC Public Health 2020;20:428. 32238165 10.1186/s12889-020-08518-7 PMC7115083

[27] Pronk NP. Physical activity promotion in business and industry: evidence, context, and recommendations for a national plan. J Phys Act Health 2009;6:S220–35. 20120131

[28] Pronk NP. Implementing movement at the workplace: approaches to increase physical activity and reduce sedentary behavior in the context of work. Prog Cardiovasc Dis 2021;64:17–21. 33164840 10.1016/j.pcad.2020.10.004

[29] Munn Z , Peters MDJ, Stern C, et al Systematic review or scoping review? Guidance for authors when choosing between a systematic review or scoping review approach. BMC Med Res Methodol 2018;18:143. 30453902 10.1186/s12874-018-0611-x PMC6245623

[30] Arksey H , O'Malley L. Scoping studies: towards a methodological framework. Int J Soc Res Methodol 2005;8:19–32.

[31] Tricco AC , Lillie E, Zarin W, et al PRISMA extension for scoping reviews (PRISMA-ScR): checklist and explanation. Ann Intern Med 2018;169:467–73. 30178033 10.7326/M18-0850

[32] Booth A. Clear and present questions: formulating questions for evidence based practice. Library Hi Tech 2006;24:355–68.

[33] Guest G , MacQueen K, Namey E. Applied Thematic Analysis. Thousand Oaks, CA: SAGE, 2012.

[34] Graneheim UH , Lindgren BM, Lundman B. Methodological challenges in qualitative content analysis: a discussion paper. Nurse Educ Today 2017;56:29–34. 28651100 10.1016/j.nedt.2017.06.002

[35] Sparkes A , Smith B. Qualitative Research Methods in Sport, Exercise and Health: From Process to Product. London, UK: Routledge, 2014.

[36] Pronk NP , Faghy MA. Causal systems mapping to promote healthy living for pandemic preparedness: a call to action for global public health. Int J Behav Nutr Phys Act 2022;19:13. 35130923 10.1186/s12966-022-01255-7 PMC8822751

[37] Paez A. Grey literature: an important resource in systematic reviews. J Evid Based Med 2017;10:233–40. 28857505 10.1111/jebm.12266

