# Supplementary material for: Workplace physical activity practices in real life: a scoping review of grey literature for small- and medium-sized enterprises
Source: Eur J Public Health. 2022 Aug 26;32(Suppl 1):i22–7. doi: 10.1093/eurpub/ckac083 (PMC9421405; doi:10.1093/eurpub/ckac083)
Supplement: ckac083_Supplementary_Data [file ckac083_supplementary_data.zip › ckac083-suppl-data/APPENDIX 1.docx]

**APPENDIX 1.** Flowchart diagrams of the systematic Google search in the Catalan, Finnish, French, Dutch, English and Spanish languages.

**Flowchart diagram of the systematic Google search in the Spanish and Catalan languages:** Spanish and Catalan Google search strategies yielded 213,983 records and 3,534 respectively. The first 100 results of each language were assessed and analyzed and the final number of included documents or files were 10 in Spanish and 3 in Catalan.

Records identified through database searching via the 17 **SPANISH** search strings in google

(n = 213.983)

Records screened by title

(=first 10 google pages if there were 10)

(n = 10)

Full-text documents assessed for eligibility

(n = 10)

Documents included in qualitative synthesis

(n = 10)

Records identified through database searching via the 17 **CATALAN** search strings in google

(n = 3.534)

Records screened by title

(=first 10 google pages)

(n = 3)

Full-text documents assessed for eligibility

(n = 3)

Documents included in qualitative synthesis

(n = 3)

**Flowchart diagram of the systematic Google search in the Finnish language.**

The Finnish Google search strategy yielded 157 records. After checking the first 100 results, none of those were included for data extraction and analysis. Six documents or files, identified through other manual sources, were included for data extraction and analysis

Records identified through database searching
(n = 157 )

Additional records identified through other sources
(n = 6 )

Documents included in qualitative synthesis
(n = 6 )

Full-text documents excluded, with reasons
(n = 0 )

Full-text document assessed for eligibility
(n = 0 )

Records excluded
(n = 100 )

Records screened
(n = 100 )

Records after duplicates removed
(n = 100 )

**Flowchart diagram of the systematic Google search in the French and Dutch languages.**

The French and Dutch Google search strategy yielded 16.320.780 records in French and 282.744 in Dutch.  The first 100 results of each language were analysed and the final number of included documents or files was 23 in French and 5 in Dutch.

Records identefied thourgh database searching via the 40 **FRENCH** searchstrings in google

(n = 16.320.780)

Records screened by title

(=first 10 google pages if there were 10)

(n = 3.900)

full-text documents assessed for eligibility

(n = 63)

Documents included in qualiteve synthesis

(n = 8)

Totale of the companies with intervetnions in the included documents

(n = 23)

Records identefied thourgh database searching via the 40 **DUTCH** searchstrings in google

(n = 282.744)

Records screened by title

(=first 10 google pages)

(n = 2.702)

Full-text documents assessed for eligibility

(n = 21)

Documents included in qualiteve synthesis

(n = 3)

Totale of the companies with intervetnions in the included documents

(n = 5)

F**lowchart diagram of the systematic Google search in the English language.**

Documents included in qualitative synthesis
(n = 129)

Full-text document assessed for eligibility
(n = 131)

Full-text documents excluded, with reasons
(n = 2)

Records screened
(n = 273 )

Records after duplicates removed
(n = 273 )

Records excluded
(n = 142)

The English Google search strategy yielded a total of 883.717 records. The first 100 results were assessed and analyzed, resulting in the inclusion of 226 documents or file for data extraction and analysis.

Additional records identified through other sources
(n = 57 )

Records identified through database searching
(n = 226 )
