# Supplementary figures and images for: Workplace physical activity practices in real life: a scoping review of grey literature for small- and medium-sized enterprises
Source: Eur J Public Health. 2022 Aug 26;32(Suppl 1):i22–7. doi: 10.1093/eurpub/ckac083 (PMC9421405; doi:10.1093/eurpub/ckac083)

## Slide 1
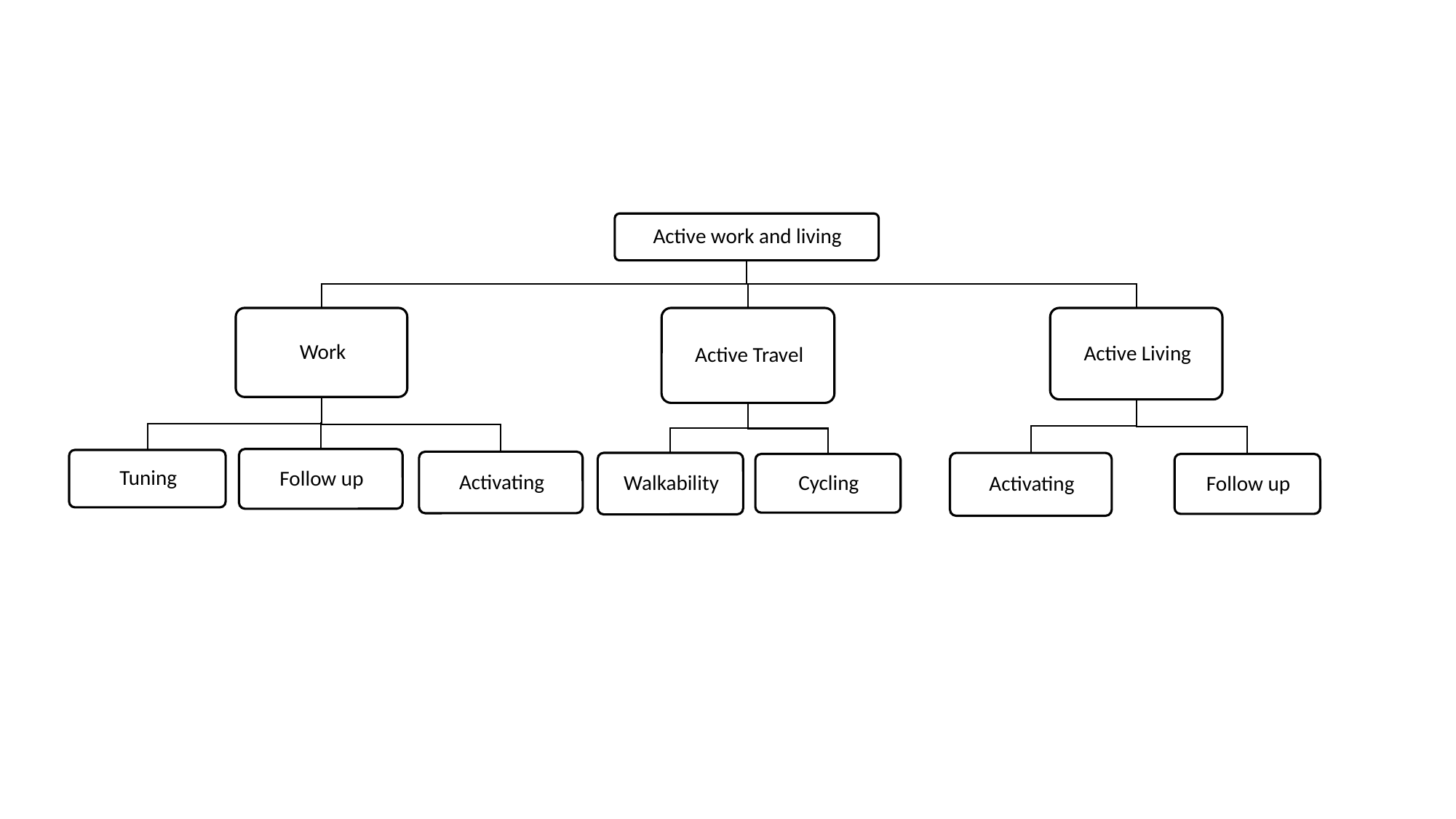

## Slide 2
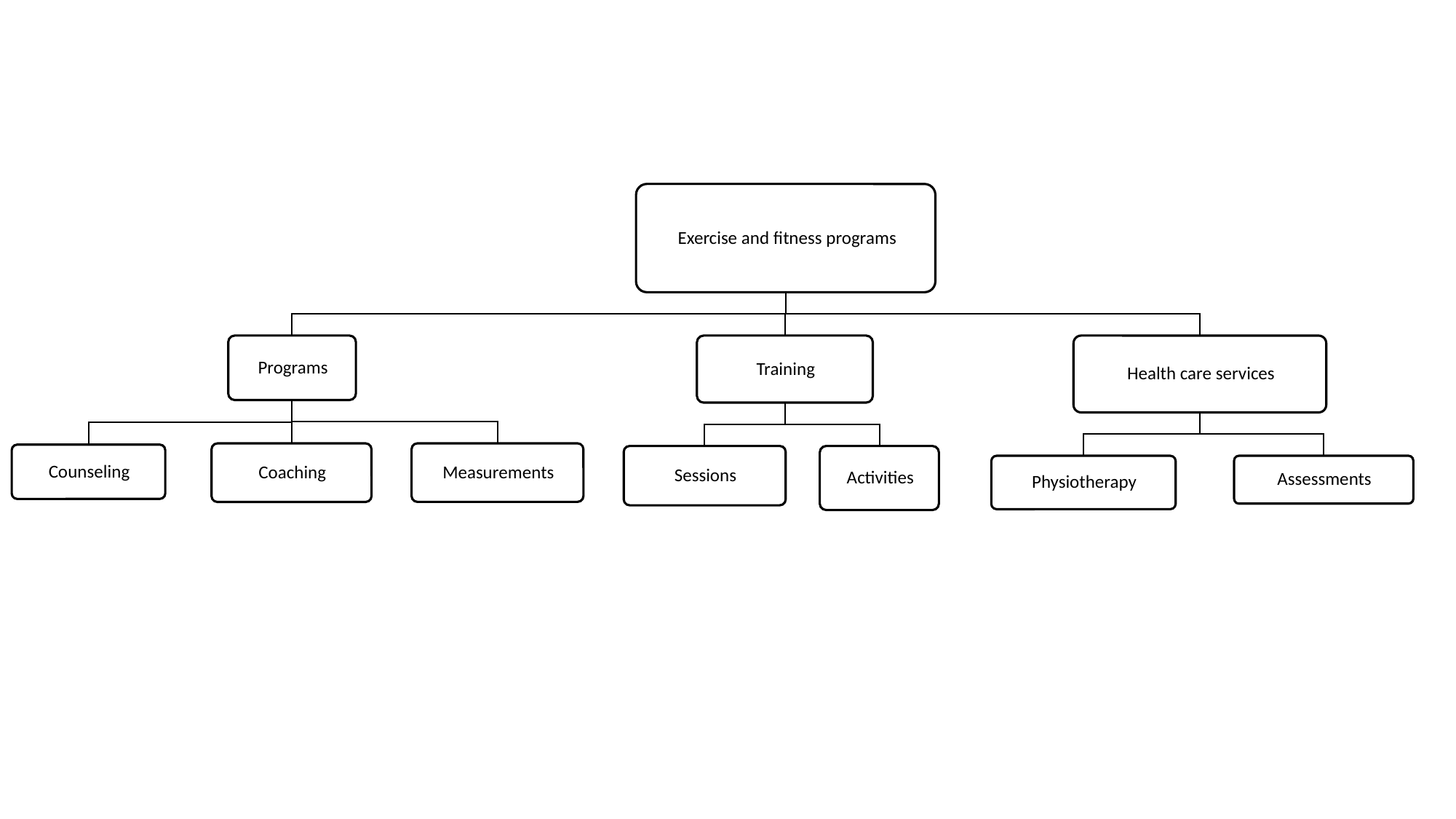

## Slide 3
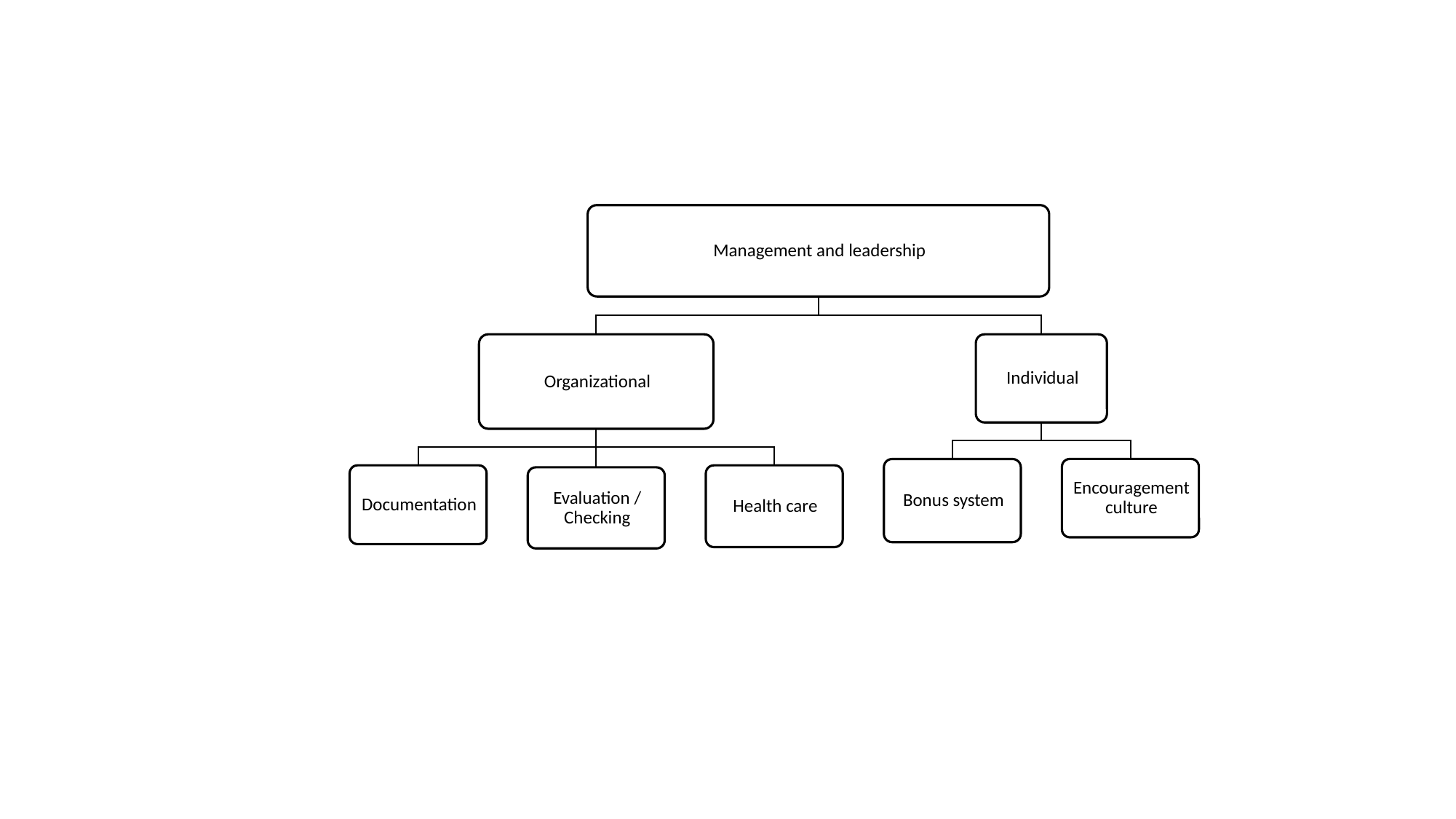

## Slide 4
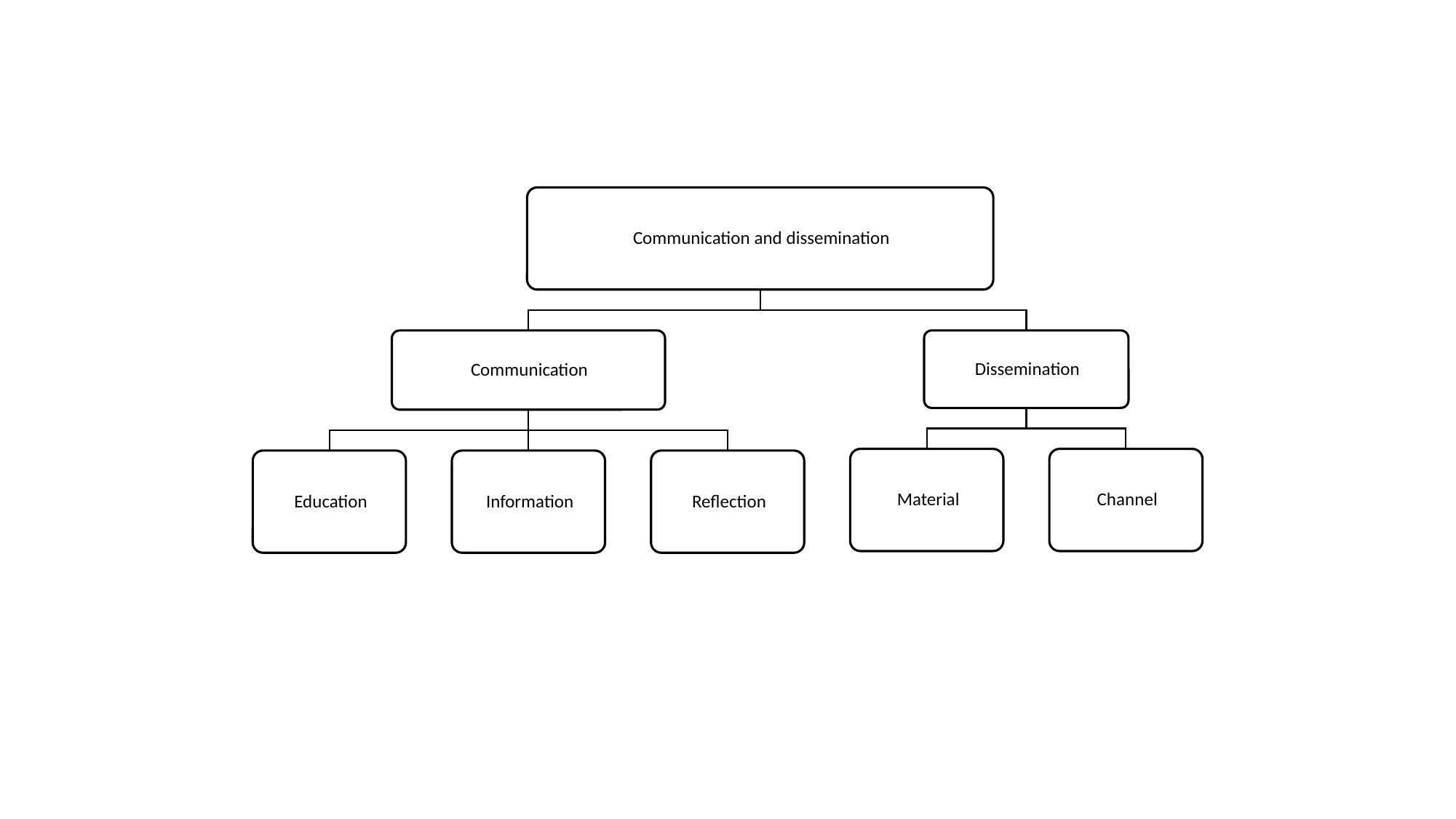

## Slide 5
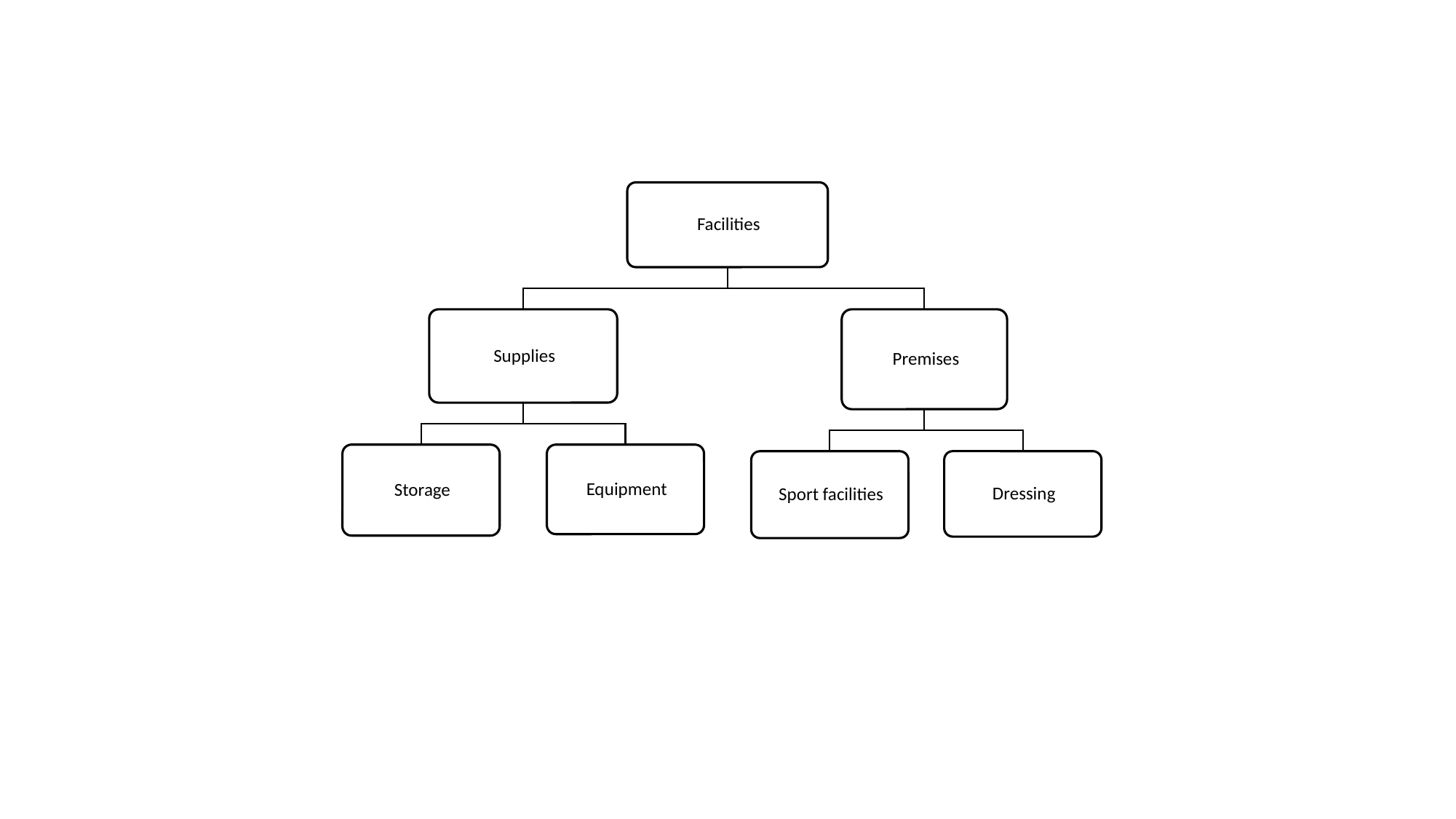

Supplement: ckac083_Supplementary_Data [file ckac083_supplementary_data.zip › ckac083-suppl-data/Supplementary Figures_Feature trees.pptx]
